# Transcriptome Analysis Identifies Candidate Genes and Functional Pathways Controlling the Response of Two Contrasting Barley Varieties to Powdery Mildew Infection

**DOI:** 10.3390/ijms21010151

**Published:** 2019-12-24

**Authors:** Yingbo Li, Guimei Guo, Longhua Zhou, Yunyun Chen, Yingjie Zong, Jianhua Huang, Ruiju Lu, Chenghong Liu

**Affiliations:** Biotech Research Institute, Shanghai Academy of Agricultural Sciences/Key Laboratory of Agricultural Genetics and Breeding, Shanghai 201106, China; liyingbo163@163.com (Y.L.); guo_gm@126.com (G.G.); zhoulonghua@saas.sh.cn (L.Z.); cyy_082@163.com (Y.C.); 18916925917@163.com (Y.Z.); sw1@saas.sh.cn (J.H.)

**Keywords:** powdery mildew, barley, plant defense, transcriptome analysis

## Abstract

Powdery mildew caused by *Blumeria graminis* f. sp. *hordei* (*Bgh*) is one of the most serious diseases in barley. The numerous barley varieties across China provide valuable genetic resources to screen the resistant germplasm and to discover the primary genes of resistance to powdery mildew. In this study, Chinese barley variety Feng 7 was identified as a highly resistant genotype which limited *Bgh* colonization by cell apoptosis using leaf staining assay, while another variety Hua 30 showed high susceptibility. The performance of high resistance to *Bgh* in F_1_ plants from the two varieties suggested dominant gene(s) controlled the resistance to powdery mildew in Feng 7. To understand the host transcriptional response to *Bgh* infection, these two barley varieties Feng 7 and Hua 30 were inoculated with *Bgh*, and their transcriptional profiling using RNA sequencing (RNA-seq) at four time points (12 h post-inoculation (hpi), 24 hpi, 48 hpi, and 72 hpi) were compared. 4318 differentially expressed genes (DEGs), including 2244 upregulated and 2074 downregulated genes, were detected in Feng 7, compared with Hua 30 at 12 hpi. 4907 DEGs (2488 upregulated and 2419 downregulated) were detected at 24 hpi. 4758 DEGs (2295 upregulated and 2463 downregulated) were detected at 48 hpi. 3817 DEGs (2036 upregulated and 1781 downregulated) were detected at 72 hpi. The results showed the number of DEGs between two varieties peaked at 24 hpi (for the upregulated) or 48 hpi (for the downregulated), which is matched with the processing of *Bgh* infection. In addition, the number of upregulated DEGs involved in the functional pathways of plant defense (mitogen-activated protein kinase (MAPK) pathway and plant hormone signal transduction) is elevated remarkably at 24 hpi. Six candidate genes (*PR13*, *glutaredoxin*, *alcohol dehydrogenase,* and *cytochrome P450*) were identified in Feng 7. All of them present continuous expression at higher levels upon *Bgh* infection, compared with the performance in Hua 30, which revealed the potential contribution to Feng 7 mediate resistance to *Bgh*. In conclusion, the candidate genes and relevant pathways provided key information towards understanding the defense of barley to *Bgh* attack and the molecular mechanisms of different genetic resistance to powdery mildew.

## 1. Introduction

Powdery mildew caused by *Blumeria graminis* f. sp. *hordei* (*Bgh*) is one of the most important diseases in barley and leads to substantial yield loss every year [1]. The development and utilization of resistant cultivars has been recognized as one of the most economical, environmentally safe, and effective strategies for disease control. The novel germplasms with high resistance and a more comprehensive understanding of molecular mechanisms of *Bgh* resistance are crucial for the breeding of resistant cultivars.

Plants possess remarkably sophisticated mechanisms to defend themselves against attacks by pathogens. The first line is categorized into basal resistance triggered by the perception of pathogen-associated molecular patterns (PAMPs), always represented by the ion fluxes across the plasma membrane, oxidative bursts, and activation of mitogen-activated protein kinases. At a later stage, in order to overcome PAMP-triggered resistance, pathogens secrete small unique protein molecules (effectors), and plants develop resistance (R) gene to recognize effectors and acquire R-gene-mediated resistance, which involves hypersensitive cell death to restrict further penetration of infected pathogen [2]. Genetic resistance in barley to *Bgh* is commonly classified into two types, race-specific and non-race-specific [3]. Race-specific resistance is conferred by the interactions of resistance (R) gene products in the host, such as those encoded by the complex *Mlx* genes. The non-race-specific resistance mediated by the *mlo* gene confers broad spectrum resistance to *Bgh*. A few resistance genes against powdery mildew have been identified in barley, such as *Mla1*, *Mla6*, *Mla7*, *Mla10*, *Mla12*, *Mla13*, and *mlo* [4]. The *Mla* product recognizes unique fungal determinants by cognate fungal avirulence (Avr) genes in races of the fungus. Many *Mla* genes require *Rar1* and *Rar2* for their function, while some appear to have different signaling requirements, like *Mla1* [5]. *mlo*, based on recessive loss-of-function alleles (e.g., *mlo5*) of the *Mlo* gene, confers a broad spectrum resistance to almost all isolates of *Bgh* [6]. In addition to resistance mechanisms, antimicrobial peptides (e.g., PR 12 and PR 13) can inhibit the progression of pathogen invasion, such as membrane destabilization, interference with transport, and inhibition of protein function [7]. The peptides are fundamentally conserved in plants [8].

To date, the resistance mechanisms for powdery mildew in barley are less reported. The next-generation sequencing (NGS) technology, which emerged as a cutting-edge approach for high-throughput sequence determination, has promptly improved the efficiency of gene discovery and dramatically reduced the time, labor, and cost. The current reference genome assemblies with high quality for barley [9] provide a fundamental resource to investigate the genetic background of barley–*Bgh* resistance. Caldo et al. [10] showed that barley triggers nonspecific defense responses at the early stages of *Bgh* infection (0–16 h post-inoculation (hpi)). The barley R proteins (e.g., *Mla*) recognize the avirulence effectors at the later stages of fungal infection (16–32 hpi), which sustains the level of nonspecific defense responses and triggers the accumulation of another layer of defense responses and subsequent disease resistance. Therefore, it is important to focus on the later stages for the analysis of R-gene-mediated defense responses, and a comparison of transcriptional profiling of barley varieties with the contrasting disease resistance will provide comprehensive understanding of the response of resistant varieties to *Bgh* infection and improve the breeding of resistant varieties for better control of *Bgh*.

In our previous study, hundreds of Chinese barley varieties were used to determine the resistance level (data was not shown). Among these varieties, Feng 7 was the only variety with high resistance to local mixed *Bgh* races. The variety’s female parent is Feng 6, a variety with high resistance to *Bgh*, which was developed from a French barley line AT-1. The male parent is S500, a barley variety introduced from Mexico, with moderate resistance to *Bgh*. The present study was aimed to develop better understanding regarding the molecular mechanism of resistance conferred by Feng 7 against *Bgh*. For this purpose, reciprocal crosses were performed between Feng 7 and a susceptible variety Hua 30. It was observed that the necrotic spots gradually increased upon the *Bgh* infection by trypan blue dye exclusion staining of leaves from Feng 7, and all F1 plants presented high resistance to powdery mildew by *Bgh* inoculation test. RNA-seq data revealed that functional pathways of plant defense are consequently involved in producing cell death and plant defensin. Our report provides additional evidences for understanding the molecular mechanism with resistance to powdery mildew from Feng 7.

## 2. Results

### 2.1. Resistance Determination of Barley Variety Feng 7 to Bgh Infection

**Figure 1:**
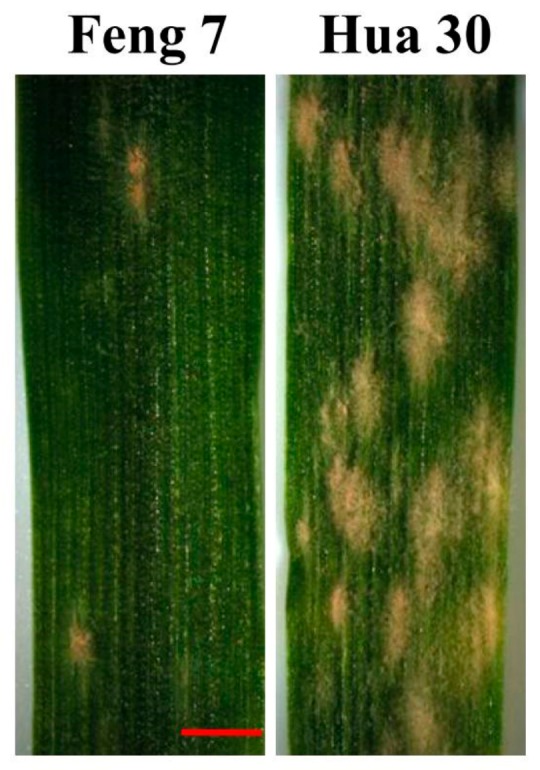
Evaluation of powdery mildew resistance of Feng 7. Hua 30 were used as susceptible controls with more conidia. Bars = 250 mm.

Leaves of Feng 7 seedlings were inoculated with local mixed *Bgh* races (Feng 7 was highly resistant to the mixed *Bgh* races, none specific to the *Bgh* race in our lab) to determine the resistance level. At 10 dpi (days post-inoculation) of *Bgh*, it was observed that very little colonies appeared on the leaves of Feng 7, while numerous colonies spread on those of Hua 30 (used as control) (Figure 1). The performance of seedling incubation assay showed high resistance to the mixed *Bgh* races, while the local variety Hua 30 was susceptible.

**Figure 2:**
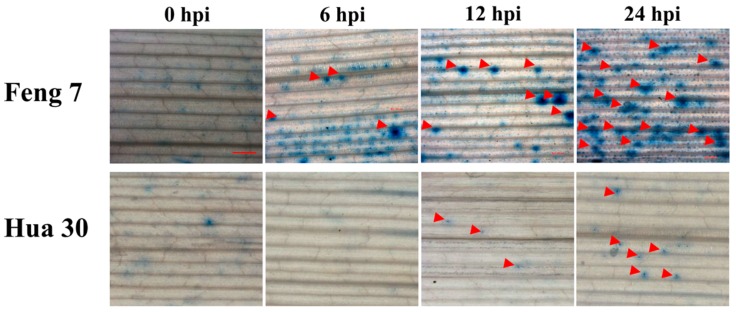
Necrotic spot detected in Feng 7 leaves at different time points upon *Blumeria graminis* f. sp. *hordei (Bgh)* infection. The necrotic spots are indicated by red arrows. Hua 30 were used as susceptible controls with less necrotic spots. Bars = 1 mm.

Trypan blue dye exclusion staining is one of the traditional methods for cell viability analysis [11]. To detect whether Feng 7 has cell apoptosis during *Bgh* infection, we performed trypan blue staining analysis at four infection stages, including 0 hpi (hours post-inoculation), 6 hpi, 12 hpi, and 24 hpi. As shown in Figure 2, it was found that the number of necrotic spots on the leaves of Feng 7 gradually increased within the progress of *Bgh* infection (Figure 2, Table 1). However, the susceptible variety Hua 30 showed very few necrotic spots (Figure 2). This indicated that Feng 7 was able to limit *Bgh* colonization by cell death.

Reciprocal crosses between Feng 7 and Hua 30 were used to analyze the resistance of genetic traits. When inoculated with *Bgh*, it was found that all the F_1_ plants showed high resistance to powdery mildew (Table 2). The finding suggested that the resistance to powdery mildew in Feng 7 was governed by dominant gene(s).

### 2.2. Illumina Sequence Analysis and Validation of Selected Differentially Expressed Genes (DEGs) Using qRT-PCR

To obtain the high quality of transcriptome data from the leaves infected by *Bgh* for both Feng 7 (resistant) and Hua 30 (susceptible), leaves of the two varieties were harvested at 12 hpi, corresponding to the recognition and prepenetration phase; 24 hpi, corresponding to haustorium establishment; and 48 and 72 hpi, corresponding to biotrophic fungal development. Twenty-four digital gene expression (DGE) libraries were constructed using Illumina sequencing platform (Supplementary Table S1). On average, 49.42 million raw tags were generated from each library. After removing the low-quality reads, total tag numbers per library ranged from 45.45 million to 47.59 million. The *Phred* value > 30 (Q30) of each library ranged from 90.81% to 93.78%, and the GC percentage of each library ranged from 54.21% to 56.40% (Supplementary Table S1). The transcriptome data of all samples was qualified to be used for further analysis of differentially expressed genes (DEGs).

**Figure 3:**
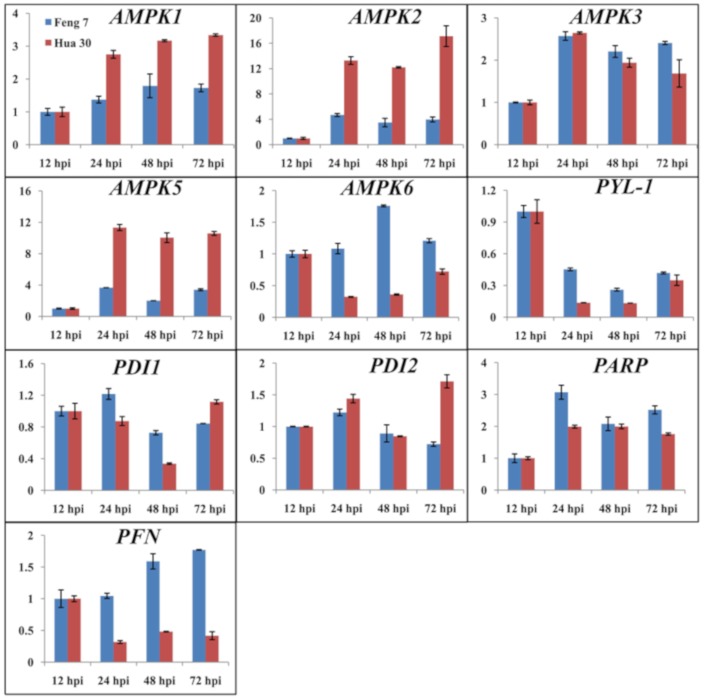
The relative gene expression of 10 randomly selected genes examined by quantitative real-time PCR. The expression levels on the y axis were relative to the 12 h post-inoculation (hpi) after normalization with the barley *actin* gene in Feng 7 and Hua 30, respectively. Data represent the mean ± SE (n = 3); hpi: hours post-inoculation.

**Figure 4:**
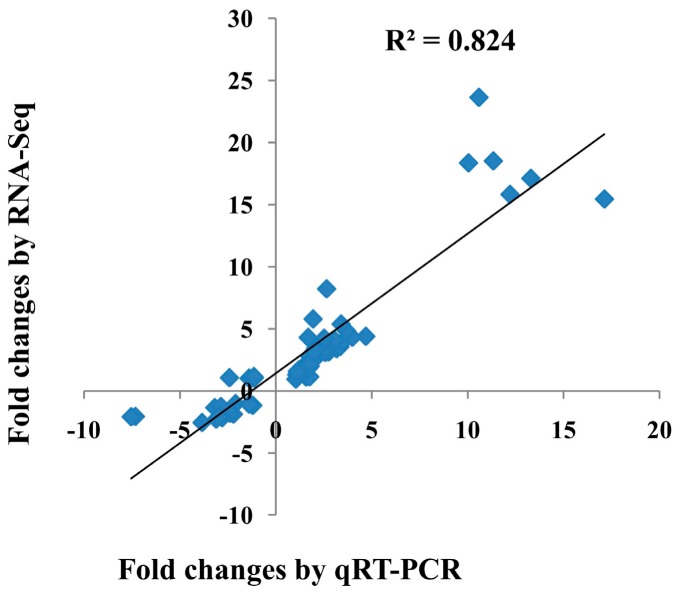
The correlation between the transcriptional changes of 10 differentially expressed genes (DEGs) by qRT-PCR and by RNA-seq. The Pearson correlation coefficient (R^2^) was 0.824.

To validate the results of the gene expression from RNA-seq data, 10 DEGs were selected for qRT-PCR analysis (Figure 3). There was a good concordance (R^2^ = 0.824) between RNA-seq data and qRT-PCR analysis (Figure 4), indicating that the gene expression levels by DGE analysis were reliable.

### 2.3. Comparative Analysis of Feng 7 and Hua 30 Transcriptomes

**Figure 5:**
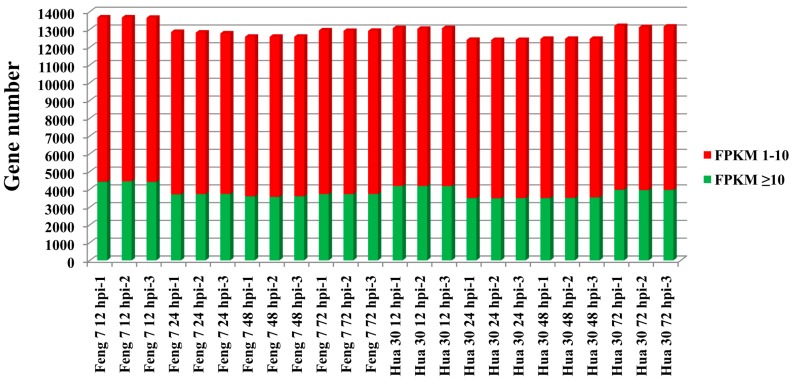
Whole genomic gene expression for Feng 7 and Hua 30 inoculation with *Bgh* at 12 hpi, 24 hpi, 48 hpi, and 72 hpi. The y axis represents gene number. Red box means fragments per kilo bases per million reads (FPKM) 1–10, green box means FPKM ≥ 10.

Whole genomic gene expression of Feng 7 and Hua 30 at four time points were compared. Analysis results showed the average expressional intensity (fragments per kilo bases per million reads (FPKM) > 1) of whole genomic genes expressed at the highest level at 12 hpi than that at other time points for both Feng 7 and Hua 30 (Figure 5).

**Figure 6:**
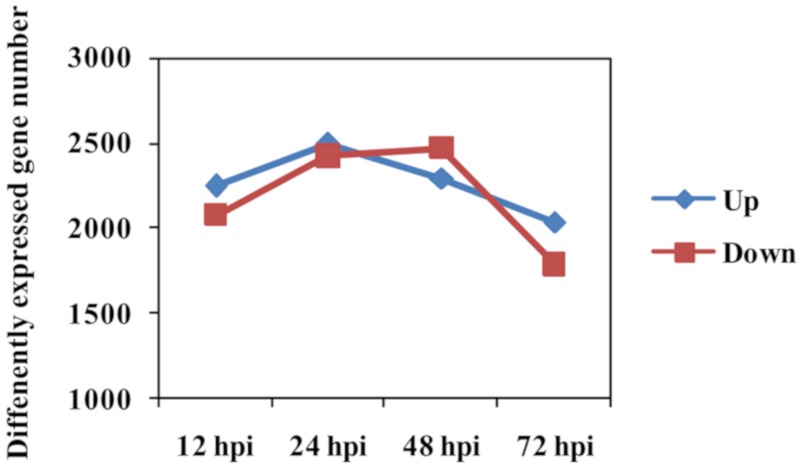
Number of differently expressed genes in Feng 7 compared with Hua 30 at four time points (12, 24, 48, and 72 hpi). The y axis represents DEGs number. Number of DEGs was counted with the criteria *p* < 0.05 and log2 (fold change) > 1.

Number of DEGs between Feng 7 and Hua 30 (used as control) was counted at each time point. The expressional pattern of DEGs was displayed as the upregulated group and the downregulated group (Figure 6). The number of the upregulated group increased from 12 hpi to 24 hpi, reached a peak at 24 hpi, and then deceased from 24 hpi to 72 hpi. The number of the downregulated group increased from 12 hpi to 48 hpi, reached a peak at 48 hpi, and then decreased from 48 hpi to 72 hpi.

**Figure 7:**
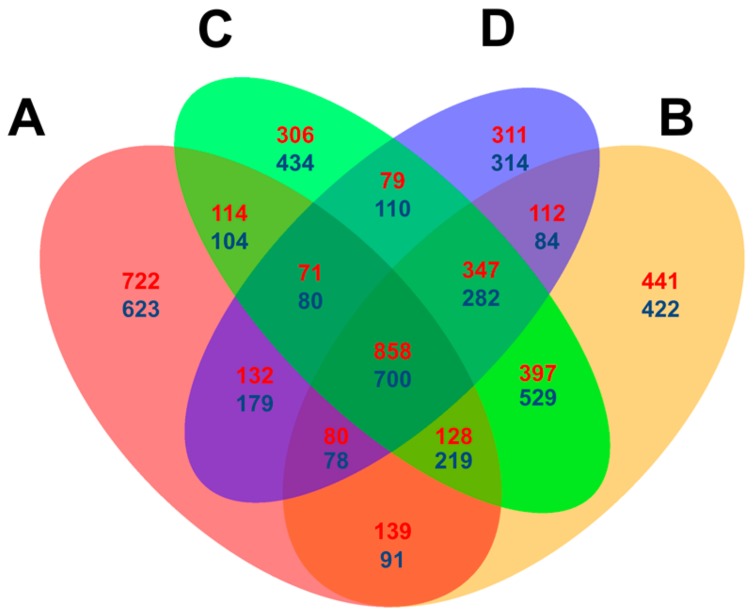
Venn diagram comparison of differentially expressed genes (assigned by *p* < 0.05 and log2 (fold change) > 1) at four infection stages (12, 24, 48 and 72 hpi) in Feng 7 compared with Hua 30. A: DEGs at 12 hpi; B: DEGs at 12 hpi; C: DEGs at 48 hpi; D: DEGs at 72 hpi. Red highlighted numbers represent the amount of upregulated DEGs, and blue highlighted numbers represented the amount of downregulated DEGs.

Venn diagrams were constructed using the DEGs counted at each time point with the criteria [=(*p* < 0.05, log2 (fold change) > 1) to compare the expressional level difference between Feng 7 and Hua 30 (used as control). In summary, 722 up- and 623 downregulated DEGs were specifically detected at 12 hpi, which is higher than the numbers (ranging from 306 to 434) detected specifically at other time points. In total, 858 up- and 700 downregulated DEGs were detected at all four time points (Figure 7). The results indicate that some DEGs are dependently regulated on the infection time, and some are independently regulated.

### 2.4. Gene Ontology (GO)-Based Analysis of the DEGs

**Figure 8:**
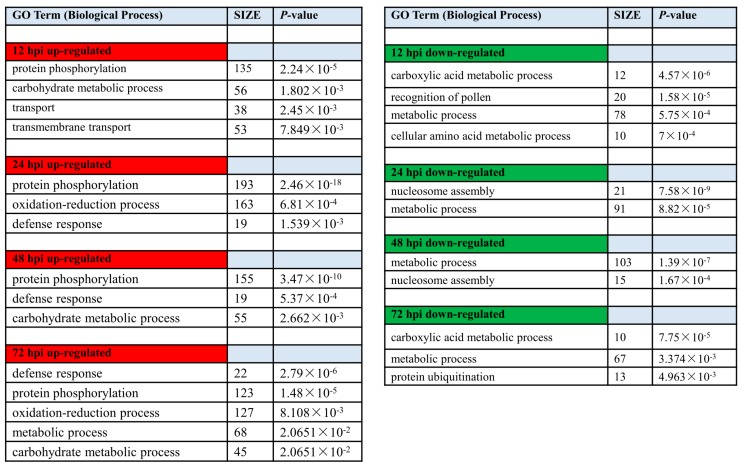
Gene ontology enrichment of DEGs (biological process) in Feng 7 compared with Hua 30 under *Bgh* infection at 12, 24, 48, and 72 hpi. Number of DEGs contained in respective categories is given as SIZE. Only categories with SIZE larger than 10 and *p* < 0.05 are displayed. Red color show up-regulated biological process, green color show down-regulated biological process.

Functional gene categories (GO terms) were identified using gene set enrichment analysis, to detect the significant biological processes in Feng 7 compared with Hua 30 under *Bgh* infection (Figure 8, Supplementary Data S1 and S2). The functional category significantly over-represented among upregulated was protein phosphorylation at all four time points. The category oxidation-reduction process was over-represented at 24 hpi and 72 hpi. The category defense response was over-represented from 24 hpi to 72 hpi. Meanwhile, the categories metabolic process, cellular amino acid metabolic process, and nucleosome assembly were overrepresented among downregulated transcripts. This suggests more genes related to signal transduction and stress response in Feng 7 were upregulated under *Bgh* infection.

### 2.5. KEGG Pathway Analysis of the DEGs

To identify the main metabolic pathways related to plant defense for the DEGs detected in Feng 7 infected by *Bgh*, Kyoto Encyclopedia for Genes and Genomes (KEGG) enrichment analysis was used, and 11 predicted pathways were mapped (listed in Table 3; their corresponding IDs are provided in Supplementary Data S3 and S4). A total of 99 upregulated genes and 64 downregulated DEGs were mapped with the metabolic pathways related to plant defense at 24 hpi, which is a key time point for the most up-/downregulated DEGs detected at this point. In addition, more DEGs were upregulated across the time course upon the *Bgh* infection.

For DEGs in galactose metabolism, ABC transporters, brassinosteroid biosynthesis, plant hormone signaling, calcium signaling pathway, mitogen-activated protein kinase (MAPK) signaling, and steroid hormone biosynthesis, more DEGs were upregulated upon *Bgh* infection. For DEGs in protein processing in endoplasmic reticulum, photosynthesis-antenna proteins, glutathione metabolism, and phenylalanine metabolism, more DEGs were downregulated upon *Bgh* infection.

### 2.6. Clustering Analysis of DEGs to Compare Gene Expression Patterns

It seems that not all changes in gene expression are expected to be direct consequences of pathogen infection. To identify coregulated transcripts during *Bgh* infection in Feng 7, the statistical analysis used in this study was focused on the overall pattern of expression based on the kinetics of infection. Short Time-series Expression Miner (STEM) clustering and GO enrichment analysis of the resulting clusters were performed in Feng 7 to compare with Hua 30 across four inoculation time points.

**Figure 9:**
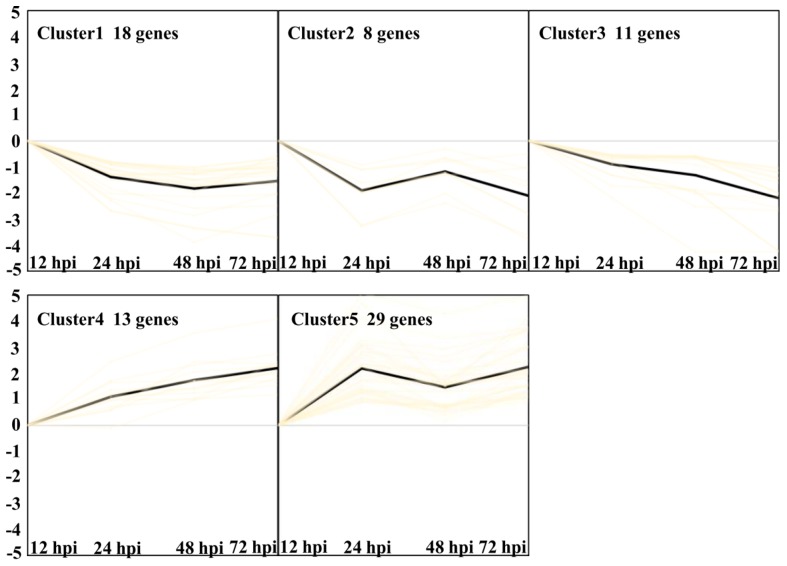
Overview of 5 different clusters of *Bgh* regulated transcripts at four infection stages (12, 24, 48, and 72 hpi). Using the *k*-means clustering algorithm, five clusters were calculated based on the list of transcripts differentially expressed. Prior to cluster analysis, 12 hpi was set as control, and the data were standardized. Each diagram shows the relative transcript levels at 24, 48, and 72 hpi, respectively. Number of genes contained in each cluster is given within the respective diagram.

Finally, five clusters were found with the criteria (*p* < 0.05, Figure 9, Supplementary Data S5) [12]. Clusters 1, 2, and 3 represent transcripts expressed downregulated from 24 hpi to 72 hpi. Clusters 4 and 5 represent transcripts expressed upregulated from 24 hpi to 72 hpi. GO enrichment analysis of Cluster 1 showed a significant over-representation of the function category termed transmembrane transport with four genes. Cluster 2 showed three genes, which were termed carbohydrate metabolic process. Six genes in Cluster 3 were termed metabolic process. Cluster 4 showed two significant function categories, termed as defense response and oxidation-reduction process with three genes, respectively. Cluster 5 showed a significant over-representation of the function category termed oxidation-reduction process with 12 genes. The results indicated that oxidation-reduction and defense response may be important biological processes related to Feng 7-mediated resistance.

### 2.7. Identification of Candidate Genes that Were Related to Feng 7 Mediated Resistance

According to Caldo et al. [10], the existence of host R proteins will trigger the accumulation of another layer of defense-related transcripts at the later stages (post-16 hpi) of fungal infection, so we restricted our attention to DEGs which were upregulated in Feng 7 from 24 hpi (FPKM > 1 and log2 (fold change) > 1 from 24 hpi to 72 hpi) (Supplementary Table S3). The DEGs which are downregulated in Feng 7 from 24 hpi (FPKM > 1 and log2 (fold change) <−1 from 24 hpi to 72 hpi) were further identified (Supplementary Table S4). This strategy may have missed some interesting genes, but this was counterbalanced by the reliability of digging targeted genes specific to the resistance.

**Figure 10:**
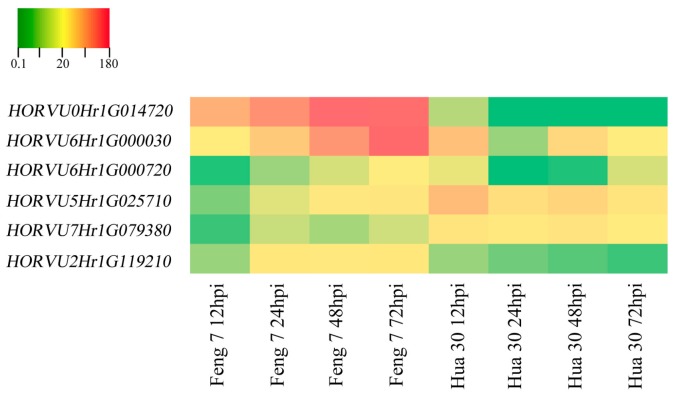
Heat maps of the six identified genes in Feng 7 and Hua 30 at four infection stages (12, 24, 48, and 72 hpi), using all 12 hpi samples as controls. The bottom color bar represents the log2 of FPKM for each gene, ranging from green (0.1) to orange (180). A deeper color indicates more transcript accumulation.

**Figure 11:**
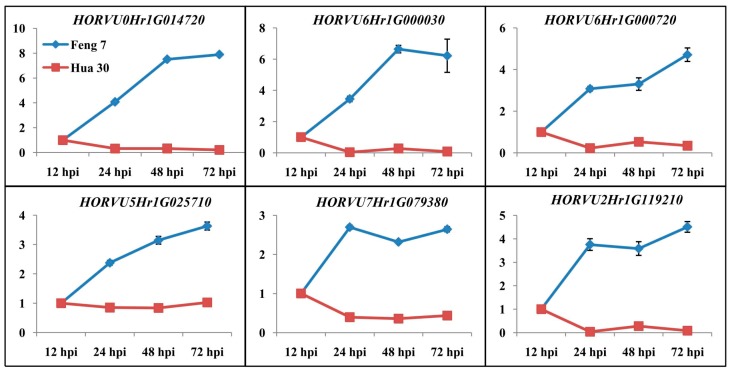
Validation of DGE transcriptome results by qRT-PCR of the six genes in Feng 7 and Hua 30 after inoculation with *Bgh*. The expression levels on the y axis were relative to 12 hpi after normalization with the barley *actin* gene in Feng 7 and Hua 30, respectively. The data are presented as average ± SD with n = 3.

Six candidate upregulated genes were identified following the strategy (Supplementary Table S3). These genes include 3-*thionin*, a glutaredoxin, an *alcohol dehydrogenase* and a *cytochrome P450* protein. Heat maps of these six genes were constructed in Feng 7 and Hua 30 at four inoculation time points (Figure 10). They display a clear trend that the expression of six genes was significantly increased in Feng 7, while most of these genes’ expression was significantly decreased in Hua 30. These results were further confirmed by the qRT-PCR analysis (Figure 11).

## 3. Discussion

In this study, it was observed that the number of necrotic spots was gradually increased with the time course upon *Bgh* infection in barley variety Feng 7. Cell apoptosis was one of the important cellular defense reactions of plants to pathogens [13] and was a very late feature of the disease course [2,3]. This suggests that cell apoptosis is a critical process for the resistance of Feng 7 to powdery mildew. The uniformed resistance in F_1_ population from the reciprocal crosses between Feng 7 and a susceptible barley variety Hua 30 showed that the resistance was governed by dominant gene(s) in Feng 7. In barley, *mlo*-gene-mediated resistance leads to an immune phenotype to *Bgh* [6]. As Feng 7 was not immune to *Bgh* (Figure 1), it suggests the resistance was not mediated by the *mlo* gene. Numbers of resistance genes could be predicted based on the performance of powdery mildew resistance in F_2_ population.

Dynamic expressional patterns of DEGs were carefully examined across the four time points (12 hpi, 24 hpi, 48 hpi, and 72 hpi). The number of total DEGs, as well as the number of upregulated genes, was increased during the period from 12 hpi to 24 hpi in Feng 7. This period of massive regulation on a great number of genes is matched with the whole growth cycle of powdery mildew fungi. Starting with a conidium adhering to the barley epidermal cells, the *Bgh* needs 24 h to complete the infection and grow secondary hyphae [14]. Accordingly, the plants need to defend the first round of *Bgh* attack within 24 h. It is worth identifying the activated genes involved in the first round of defense response.

The GO analysis and metabolic pathways were enriched for all the DEGs detected in Feng 7 by comparison with Hua 30. Protein phosphorylation, oxidation-reduction process, and defense response were significantly over-represented among the upregulated categories in Feng 7. This suggested more genes participated in signal transduction, stress response, and defense reaction in Feng 7 compared with in Hua 30. Among the kinase-related pathways, MAPK pathways are highly conserved in all eukaryotes, which are involved in the regulation of diversity plant development and in responses to pathogen attack in plants, including the biosynthesis/signaling of plant stress/defense hormones, reactive oxygen species (ROS) generation, stomatal closure, defense gene activation, phytoalexin biosynthesis, cell wall strengthening, and hypersensitive response (HR) cell death [15,16]. More upregulation genes related to the MAPK pathway were detected in Feng 7 at 24 and 48 hpi (Table 3), which indicated the MAPK pathway was important for Feng 7-mediated resistance. Endoplasmic reticulum (ER) stress (also named unfold protein response (UPR)) is initiated by different types of conditions, such as biotic or abiotic stresses, as well as protein synthesis overload [17]. Protein ubiquitination plays a pivotal role in the ER stress [18]. Recently, it was found that the UPR can be elicited in the course of plant development and defense [19]. In multicellular eukaryotes, if the UPR fails, ER-stress-induced apoptosis occurs [20]. In the present study, more genes related to UPR and ER stress were downregulated in Feng 7 than in Hua 30 (Figure 8 and Table 3), and this may lead to cell apoptosis.

To identify coregulated transcripts continuously involved in the defense to *Bgh* infection, STEM clustering analysis was performed on the DEGs between Feng 7 and Hua 30 across four inoculation time points. GO enrichment analysis of the resulting clusters revealed that more upregulated genes participate in the biological processes of oxidation-reduction and defense response, and more downregulated genes participate in metabolic process. The oxidation-reduction process is one of the basic processes in plant cells and promotes molecular oxygen to produce reactive oxygen species (ROS), particularly superoxide, H_2_O_2_, and singlet oxygen [21,22]. ROS usually acts as second messenger in many biological processes associated with plant growth and development [23,24] and also contributes to programmed cell death (PCD) in plant defense response [13]. Defense responses are always associated with increased demands for energy in plant, so the plant cell reduced part of the metabolic process which related to growth, and increased metabolic process, such as defense-related protein synthesis, which related to defense response [25]. This was inconsistent with the results that the part of genes in upregulated express pattern clusters (Clusters 4 and 5) was termed *carbohydrate metabolic* (Supplementary Data S5). These suggested the plant cells organize metabolic processes in an elegant way with defense response to *Bgh* infection.

Six candidate genes involved in defense response were identified by digging the DEGs which were activated specifically in Feng 7 with continuous expression (upregulated from 24 hpi to 72 hpi). Three of the candidate genes encode thionin protein. Thionin is known as plant toxin because of its toxicity towards microbes and has been described for the PR13 group [26]. Previous research showed that thionins can be induced by infection with various microbes [27,28,29], and thionin has been shown to be related to the release of the hormone methyl jasmonate upon plant wounding or microorganism invasion [30,31]. The fourth of the candidate genes encodes glutaredoxin protein. Glutaredoxins are small heat-stable oxidoreductases that transfer electrons from glutathione to oxidized cysteine residues [32]. In *Arabidopsis*, the expression of *glutaredoxin* (*GRX480*) was induced by SA and dependent on *NPR1*. Overexpression of *GRX480* abolished methyl-jasmonate-induced *PDF1.2* expression [32]. The fifth of the candidate genes encodes alcohol dehydrogenase, which is a key enzyme responsible for catalyzing the reduction of acetaldehyde to ethanol using NADH as reductant [33]. Alcohol dehydrogenase has been reported to play roles in plant defense to pathogens. Shi et al. showed that overexpression of *alcohol dehydrogenase 1* (*AtADH1*) in *Arabidopsis* confers enhanced disease resistance against bacterial pathogen *Pst* DC3000 [34]. Rong et al. showed that overexpression of *alcohol dehydrogenase* (*TaCAD12*) contributes to resistance to the Sharp Eyespot Disease in wheat [35]. The sixth of the candidate genes encodes cytochrome P450 protein. Cytochrome P450 is involved in substrate conversion and catalysis of a variety of chemical reactions. When *CaCYP1*, a cytochrome P450 gene from chili pepper, was silenced, the plant compromised the basal pathogen defense response to *Xanthomonas axonopodis* [36]. The expression of the six genes was upregulated in Feng 7 from 24 hpi to 72 hpi, but downregulated in Hua 30 (Figure 10 and Figure 11). This suggests that these genes participate in *R*-gene(s)-mediated defense response in Feng 7.

**Figure 12:**
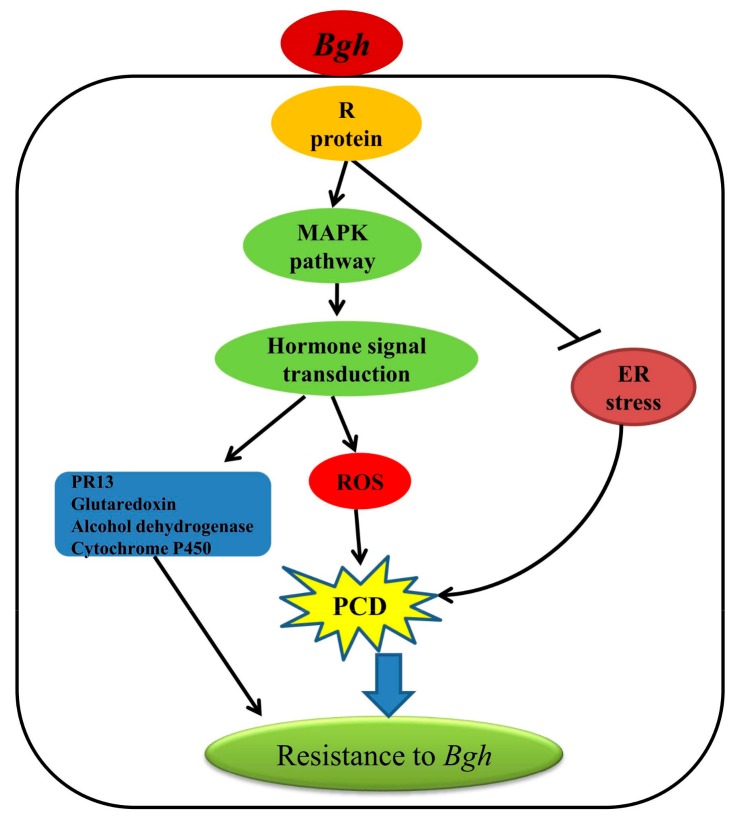
A putative network mediated by Feng 7 in regulation of the resistance to powdery mildew. The triangular arrow show promote the down stream pathways and genes, T-arrow show suppress the down stream pathways.

Our transcriptome data provided a comprehensive data set for gene expressional profiles between two barley varieties that showed contrasting resistance (highly resistant and susceptible) to powdery mildew. A putative network underlying the resistance inherited by Feng 7 was proposed (Figure 12). Upon *Bgh* infection, the MAPK pathway activated, which subsequently activated the ROS and plant hormone signal transduction pathway. Meanwhile, ER stress pathway was suppressed, which may have led to PCD. Consequently, *PR 13*, *glutaredoxin*, *alcohol dehydrogenase* and *cytochrome P450* were induced, which enhanced the plant resistance to *Bgh*. To identify the possible functional gene(s) conferred the resistance, further investigation, such as map-based cloning in F_2_ population from reciprocal crosses of Feng 7 and Hua 30, will be carried out in our lab.

## 4. Materials and Methods

### 4.1. Plant Materials, Growth Conditions, Bgh Treatment

Feng 7, a Chinese barley variety, was introduced from Dali Academy of Agricultural Sciences and Technology Extension, Yunnan Province, China. Hua 30, a popular variety cultivated in the area of Yangtze River delta of China, was grown in the farm of Shanghai Academy of Agricultural Sciences, Shanghai, China. Barley seeds were grown in the greenhouse with 22 °C and a photoperiod of 12 h. Mixed races of *Bgh* from the field of Shanghai were maintained on seedlings of a powdery-mildew-susceptible variety Hua 30 in greenhouse under a 14 h light/10 h darkness (22/18 °C, 70% humidity) regime. Two-leaf stages of Feng 7 and Hua 30 seedlings were shaken off with the mixed races of *Bgh*, and then the barley leaves were harvested at four growth stages after infection: 12 h, 24 h, 48 h, and 72 h respectively, and three biological replicates were collected for each sampled.

### 4.2. Trypan Blue Staining

Staining with trypan blue was performed essentially as described by Peterhänsel et al. [37]. The inoculated barley leaves were then cut into 1.5 cm segments at the indicated time (0, 6, 12, and 24 h), and detached leaves were stained by boiling for 8 min in alcoholic lactophenol (96% ethanol/lactophenol 1:1 (*v*/*v*)) containing 0.1 mg/mL trypan blue (Sigma, Saint Louis, MO, U.S.A.) and cleared in a chloral hydrate solution (2.5 mg/mL) overnight. The cleared leaf segments were then stored in 50% glycerol. For microscopic observation, the treated leaf segments were mounted on glass slides in 50% glycerol and examined (200×) using an Olympus microscope (Olympus, Tokyo. Japan). Four leaf pieces from each time point were observed. Each infection time point represents at least 3 leaf segments. Standard deviations and a paired sample *t*-test for statistical analysis were performed using the SAS software.

### 4.3. Evaluation of Powdery Mildew Resistance

Powdery mildew resistance of the F_1_ generation plants (Feng 7 × Hua 30 and Hua 30 × Feng 7) were evaluated by inoculation with *Bgh*. Two-leaf-stage seedlings of plants were treated with the *Bgh*, then placed on medium containing 20 mg/L 6-benzylaminopurine, and 0.6% agar. The resistance level was classified as grades 0–4 (0, 0; 1, 2, 3, and 4) as described by Sheng [38]. Grade 0 represents the highest resistance level to *Bgh*, at which leaves are immune; grade 0; represents high resistance, under which only hypersensitive mosaics are observed; and grade 4 is the most susceptible level, under which all leaves are covered with large areas of hyphae, producing large amounts of spores.

### 4.4. RNA Extraction and Preparation of cDNA Library

The total RNA of *Bgh*-infected leaf samples was extracted using TRIzol reagent (Invitrogen, Carlsbad, CA, U.S.A.) according to the manufacturer’s protocol. RNA integrity was confirmed using the 2100 Bioanalyzer (Agilent Technologies, Palo Alto, CA, U.S.A.). RNA libraries can be constructed when 28S/18S of each sample ≥ 0.7 and RIN of each sample ≥ 7. RNA libraries for transcriptome sequencing were constructed according to the Illumina RNA Seq library kit (Illumina, Inc., San Diego, CA, USA). The total RNA was digested by DNase I. Then, poly-A-containing mRNA was enriched by Oligo (dT)-attached magnetic beads, and then mRNA was randomly fragmented into small segments. The first and the second strand of cDNA were synthesized using the fragments as templates, and then end repairing was done. The ends of DNA fragments were modified and ligated with adapters, and the cleaned ligation products (300–350 bp) were enriched by the PCR (15 cycles) with random primers (random hexamers), followed by gel purification. Amplified libraries were checked by the Agilent 2100 Bioanalyzer (Agilent, Technologies, Palo Alto, CA, USA).

### 4.5. RNA Sequencing and Data Analysis

RNA sequencing was performed using Illumina HiSeq^TM^ 4000 platform (Illumina, Inc, San Diego, CA, USA) for 150 bp paired-ends sequencing in Shanghai OE Biotech Co., Ltd. Quality control (QC) was done for the raw data, which were trimmed by removing all empty and low-quality reads (Q < 30 and length < 50 bp), as well as all adaptor sequences, in order to obtain clean reads. Putative transcript annotations were identified by searching the listed annotations of high confidence (HC) genes (2016) (http://webblast.ipk-gatersleben.de/barley_ibsc/downloads/Hv_IBSC_PGSB_r1_HighConf.gtf.gz). The levels of gene expression were calculated by fragments per kilo bases per million reads (FPKM) using the reads mapped to the reference sequence. DEGs were identified using the DESeq (virsion 1.26.0, European Molecular Biology Laboratory, Heidelberg, Germany) functions estimate Size Factors and nbinom Test [39]. The resulting *p* values were adjusted using Benjamini and Hochberg’s approach for controlling the false discovery rate. Genes with an adjusted *p* value < 0.05 were set as the threshold for significantly differential expression.

GO analysis was based on the website (http://geneontology.org/). Metabolic and cellular pathways were predicted by Kyoto Encyclopedia for Genes and Genomes (KEGG) mapping [40]. STEM clustering was based on Ernst and Bar-Joseph (2006) [12]. GO, KEGG pathway enrichment analysis, and STEM clustering of DEGs were respectively performed using R based on the hypergeometric distribution (R-v3.2.0, Free Software, R Foundation).

### 4.6. Quantitative RT-PCR Analysis

To validate the expressions of DEGs, 16 identified candidate genes from the KEGG enrichment analysis were randomly selected for a qRT-PCR analysis. The sequences of these candidate DEGs were obtained from the website (http://plants.ensembl.org/Hordeum_vulgare/Info/Index), and the primer pairs were designed using Primer3 (http://www.premierbiosoft.com/) according to the reference sequences. The selected gene name and primer information are listed in Supplementary Table S2. First-strand cDNA was synthesized from about 1 µg of total RNA using Super Script^TM^ reverse transcriptase (Takara, Dalian, China). The amplification reactions were performed in the ABI 7500 fast instrument (Applied Biosystems, Carlsbad, CA, U.S.A.), and the SYBR Select Master Mix (Thermo Fisher Scientific, Waltham, MA, U.S.A.) was used following the manufacturers’ instructions. The house-keeping gene *beta-actin* in barley was used as internal control [41]. The comparative CT method (^△△^CT method) of quantification was used to quantify the relative expression of specific genes [42].

## Figures and Tables

**Table 1:** The No. of necrotic spots in the Feng 7 leaves in each *Bgh* infection time. * indicates significant differences at the level of *p* < 0.05.

Time Point	No. of Necrotic Spots/Leaf Segment	Average	*t*-Test (*p* < 0.05)
0 hpi	0	1	
	2		
	1		
6 hpi	6	6.3	*
	8		
	5		
12 hpi	12	11	*
	10		
	11		
24 hpi	22	23.3	*
	23		
	25		

**Table 2:** Powdery mildew resistance evaluation of the F_1_ hybrids plant of Feng 7 and Hua 30.

F_1_ Generation	No. of Plants	No. of Resistance Plants (Grade 0, 0; 1)	No. of High Susceptible Plants (Grade 2, 3, 4)
Feng 7 × Hua 30	25	25	0
Hua 30 × Feng 7	19	19	0

**Table 3:** DEGs enriched on the pathways related to plant defense at 12, 24, 48 and 72 hpi in Feng 7. ≥1 means upregulated gene numbers, ≤−1 means downregulated gene numbers.

Pathways	12 hpi		24 hpi		48 hpi		72 hpi	
	≥1	≤−1	≥1	≤−1	≥1	≤−1	≥1	≤−1
Galactose metabolism	14		13		15		13	
ABC transporters	8		7		8		10	7
Brassinosteroid biosynthesis	3				4			3
Plant hormone signal transduction	28		29					
Calcium signaling pathway			9	8		9		
MAPK signaling pathway			22		25			
Steroid hormone biosynthesis	7		8		7		6	
Protein processing in endoplasmic reticulum				38				28
Photosynthesis-antenna proteins		20		5			14	
Glutathione metabolism		23						18
Phenylalanine metabolism		13	11	13	8	13	9	8
**Total**	**60**	**56**	**99**	**64**	**67**	**22**	**52**	**64**

## Supplementary Materials

Supplementary materials can be found at https://www.mdpi.com/1422-0067/21/1/151/s1. Supplementary Table S1: Parameters of quality control for the reads of each sample in the RNA-seq experiment. Supplementary Table S2: Primer information for qRT-PCR. Supplementary Table S3: Information of six identified upregulated genes. Supplementary Table S4: Information of fifteen identified downregulated genes. Supplementary Data 1: Information of upregulated functional categories enriched in Feng 7 at four inoculation time points. Supplementary Data 2: Information of downregulated functional categories enriched in Feng 7 at four inoculation time points. Supplementary Data 3: Information of upregulated DEGs enriched on the pathways related to plant defense in Feng 7 at four inoculation time points. Supplementary Data 4: Information of downregulated DEGs enriched on the pathways related to plant defense in Feng 7 at four inoculation time points. Supplementary data 5: Information of clusters related to plant defense in Feng 7.

## Abbreviations

Bgh	*Blumeria graminis* f. sp. *hordei*
PAMPs	Pathogen-associated molecular patterns
(R) gene	Resistance gene
NGS	Next-generation sequencing
DGE	Digital gene expression
FPKM	Fragments Per Kilo bases per Million reads
DEGs	Differentially expressed genes
GO	Gene ontology
KEGG	Kyoto Encyclopedia for Genes and Genomes
STEM	Short Time-series Expression Miner
MAPK	Mitogen-activated protein kinase
PR	Pathogenesis-related
ROS	Reactive oxygen species
PCD	Programmed cell death
HR	Hypersensitive response
