# Supplementary material for: Transcriptome Analysis Identifies Candidate Genes and Functional Pathways Controlling the Response of Two Contrasting Barley Varieties to Powdery Mildew Infection
Source: Int J Mol Sci. 2019 Dec 24;21(1):151. doi: 10.3390/ijms21010151 (PMC6982059; doi:10.3390/ijms21010151)
Supplement: Supplementary file 1 [file ijms-21-00151-s001.zip › Supplementary Table S1.docx]

**Supplementary Table S1** Parameters of quality controlled for the reads of each sample in the RNA-seq experiment.

| Sample | Raw  Reads^a^ | Raw  Bases^b^ | Clean  Reads^c^ | Clean  Bases^d^ | Valid  Bases (%)^e^ | Q30  (%)^f^ | GC  (%)^g^ |
| --- | --- | --- | --- | --- | --- | --- | --- |
| Feng 7 12 hpi-1 | 49.42M | 7.41G | 46.60M | 6.77G | 91.31 | 91.22 | 54.50 |
| Feng 7 12 hpi-2 | 49.42M | 7.41G | 47.09M | 6.84G | 92.33 | 91.85 | 54.34 |
| Feng 7 12 hpi-3 | 48.32M | 7.25G | 45.45M | 6.59G | 90.93 | 90.81 | 54.24 |
| Feng 7 24 hpi-1 | 49.42M | 7.41G | 47.42M | 6.93G | 93.53 | 93.29 | 56.13 |
| Feng 7 24 hpi-2 | 49.42M | 7.41G | 47.40M | 6.93G | 93.48 | 93.20 | 56.15 |
| Feng 7 24 hpi-3 | 49.42M | 7.41G | 47.53M | 6.95G | 93.72 | 93.48 | 56.19 |
| Feng 7 48 hpi-1 | 49.42M | 7.41G | 47.59M | 6.96G | 93.86 | 93.53 | 56.34 |
| Feng 7 48 hpi-2 | 49.42M | 7.41G | 47.40M | 6.92G | 93.41 | 93.14 | 56.40 |
| Feng 7 48 hpi-3 | 49.42M | 7.41G | 47.45M | 6.95G | 93.70 | 93.27 | 56.37 |
| Feng 7 72 hpi-1 | 49.42M | 7.41G | 47.42M | 6.94G | 93.57 | 93.31 | 56.03 |
| Feng 7 72 hpi-2 | 49.42M | 7.41G | 47.15M | 6.88G | 92.83 | 92.70 | 55.91 |
| Feng 7 72 hpi-3 | 49.42M | 7.41G | 47.42M | 6.93G | 93.45 | 93.24 | 55.87 |
| Hua 30 12 hpi-1 | 49.42M | 7.41G | 46.89M | 6.81G | 91.88 | 91.54 | 54.30 |
| Hua 30 12 hpi-2 | 49.42M | 7.41G | 47.22M | 6.86G | 92.54 | 92.00 | 54.21 |
| Hua 30 12 hpi-3 | 49.42M | 7.41G | 46.94M | 6.81G | 91.86 | 91.52 | 54.39 |
| Hua 30 24 hpi-1 | 49.42M | 7.41G | 46.91M | 6.82G | 91.99 | 91.50 | 55.77 |
| Hua 30 24 hpi-2 | 49.42M | 7.41G | 46.94M | 6.82G | 91.95 | 91.49 | 55.57 |
| Hua 30 24 hpi-3 | 49.42M | 7.41G | 47.07M | 6.84G | 92.21 | 91.71 | 55.59 |
| Hua 30 48 hpi-1 | 49.42M | 7.41G | 47.25M | 6.91G | 93.23 | 93.41 | 55.64 |
| Hua 30 48 hpi-2 | 49.42M | 7.41G | 47.14M | 6.91G | 93.18 | 93.22 | 55.67 |
| Hua 30 48 hpi-3 | 49.42M | 7.41G | 47.28M | 6.92G | 93.32 | 93.38 | 55.65 |
| Hua 30 72 hpi-1 | 49.42M | 7.41G | 47.47M | 6.95G | 93.75 | 93.78 | 54.66 |
| Hua 30 72 hpi-2 | 49.42M | 7.41G | 47.21M | 6.90G | 93.05 | 93.37 | 54.83 |
| Hua 30 72 hpi-3 | 49.42M | 7.41G | 47.06M | 6.87G | 92.70 | 93.05 | 54.81 |

^a^ The numbers counted based on the original sequence data from each sample and tranferred into the unit of “M”

^b^ The values were got from the number of raw reads multiply their sizes and tranferred into the unit of “G”

^c^ The numbers counted based on the quality controlled sequence data from each sample and tranferred into the unit of “M”

^d^ The values were got from the number of clean reads multiply their sizes and tranferred into the unit of “G”

^e^ The validity of each data

^f^ The percentage of the bases whose “*Phred* value” > 30 in the total bases. (“*Phred* value” represents the probability of successfully detecting error bases)

^g^ The percentage of the sum of the bases “G” and “C” in the total bases
