# Supplementary material for: Transcriptome Analysis Identifies Candidate Genes and Functional Pathways Controlling the Response of Two Contrasting Barley Varieties to Powdery Mildew Infection
Source: Int J Mol Sci. 2019 Dec 24;21(1):151. doi: 10.3390/ijms21010151 (PMC6982059; doi:10.3390/ijms21010151)
Supplement: Supplementary file 1 [file ijms-21-00151-s001.zip › Supplementary Table S2.docx]

Supplementary Table S2 Primer information for qRT-PCR

| Gene | Annotation | Q-PCR primer sequence |
| --- | --- | --- |
| HORVU1Hr1G076910 | *AMPK1* | ACCTCCCTTTCCATGACCAG |
|  |  | CTGTGCAGCTGATCGTCTTC |
| HORVU2Hr1G018260 | *AMPK2* | ACAAACGGCGATACTCTGGA |
|  |  | GACGCCTGCTAATAGTTGCC |
| HORVU2Hr1G027080 | *AMPK3* | AAGACAACCCCGCAATCAAC |
|  |  | AGCTTGCCGTTGTTCTTCTG |
| HORVU3Hr1G073100 | *AMPK5* | GTTCTCCAAGGACCTCACGA |
|  |  | CCATCAACTTCTTGCGGAGG |
| HORVU4Hr1G022630 | *AMPK6* | TGCGCGATAAAGGAGATTGC |
|  |  | TGGAGCACTTCAACCATCCT |
| HORVU4Hr1G055220 | *PYL* | GAGATCCTGGACGACGAGAG |
|  |  | GTCATCGATCGTGTTACCCG |
| HORVU4Hr1G043910 | *PDI1* | TGTGGCTGACAACGTTCATG |
|  |  | GATCACAACGTCCTCTTCGC |
| HORVU5Hr1G067570 | *PDI2* | GCGCAGATGTGATGGAAGAG |
|  |  | GCTTCTCCAGATCAGCTTGC |
| HORVU1Hr1G077820 | *PARP* | CAAGATCCTCGAAGCCAACG |
|  |  | AGCTTGCCCACTCACTGTA |
| HORVU1Hr1G075570 | *PFN* | GCACGACGATGATCACACAA |
|  |  | ACCCGCTGAAGAAGAAGGAA |
| HORVU0Hr1G014720 | *PR13.1* | ATTCGACATGGGACTCTGCA |
|  |  | TCGTGGCGACTCTTATTGGA |
| HORVU6Hr1G000030 | *PR13.2* | GAGTTGCTGCAAGAACACGA |
|  |  | ACCGGATTCAGGGAGAAGAT |
| HORVU6Hr1G000720 | *PR13.3* | AAAGTTGCTGCAGGTTCACC |
|  |  | TCAGTCGCATTTGGTTCACC |
| HORVU5Hr1G025710 | *Glutaredoxin* | ACCATCGTCTCCTGTTCGTT |
|  |  | TGTAAACACGAAACGCTGCA |
| HORVU7Hr1G079380 | Alcohol dehydrogenase | TCACCTACAACTCGGTCGAC |
|  |  | TTCAGCCCGTGGTACTTCAT |
| HORVU2Hr1G119210 | Cytochrome P450 | AGCTCCCGTCAGTGTAGATG |
|  |  | TTGGAGACGTATCAAGGGCC |
| HORVU1Hr1G002840.6 | *Actin* | GACTCTGGTGATGGTGTCAGC |
|  |  | GGCTGGAAGAGGACCTCAGG |
