# Supplementary material for: Transcriptome Analysis Identifies Candidate Genes and Functional Pathways Controlling the Response of Two Contrasting Barley Varieties to Powdery Mildew Infection
Source: Int J Mol Sci. 2019 Dec 24;21(1):151. doi: 10.3390/ijms21010151 (PMC6982059; doi:10.3390/ijms21010151)
Supplement: Supplementary file 1 [file ijms-21-00151-s001.zip › Supplementary Table S3.docx]

Supplementary Table S3 Information of six identified genes.

| gene_id | description |
| --- | --- |
| HORVU0Hr1G014720 | Thionin |
| HORVU6Hr1G000030 | thionin 2.1 |
| HORVU6Hr1G000720 | thionin 2.2 |
| HORVU5Hr1G025710 | Glutaredoxin family protein |
| HORVU7Hr1G079380 | Alcohol dehydrogenase |
| HORVU2Hr1G119210 | Cytochrome P450 superfamily protein |
