# Supplementary material for: Transcriptome Analysis Identifies Candidate Genes and Functional Pathways Controlling the Response of Two Contrasting Barley Varieties to Powdery Mildew Infection
Source: Int J Mol Sci. 2019 Dec 24;21(1):151. doi: 10.3390/ijms21010151 (PMC6982059; doi:10.3390/ijms21010151)
Supplement: Supplementary file 1 [file ijms-21-00151-s001.zip › Supplementary Table S4.docx]

Supplementary Table S4 Information of fifteen identified down-regulated genes.

| gene_id | description |  |
| --- | --- | --- |
| HORVU3Hr1G012420 | tRNA-splicing endonuclease subunit Sen2-2 | |
| HORVU2Hr1G089440 | phenylalanine ammonia-lyase 2 | |
| HORVU4Hr1G076420 | NAD(P)H dehydrogenase (quinone) | |
| HORVU4Hr1G006850 | Phospholipase A2 family protein | |
| HORVU3Hr1G105920 | Amino-acid permease BAT1 homolog | |
| HORVU2Hr1G042220 | Serine/threonine-protein kinase | |
| HORVU4Hr1G082000 | Zinc transporter 2 | |
| HORVU4Hr1G084740 | sugar transporter 1 | |
| HORVU1Hr1G092310 | Glucan endo-1,3-beta-glucosidase 13 | |
| HORVU2Hr1G084130 | malate synthase | |
| HORVU5Hr1G072780 | tonoplast dicarboxylate transporter | |
| HORVU2Hr1G040800 | Solute carrier family 22 member 3 | |
| HORVU7Hr1G015380 | receptor kinase 3 | |
| HORVU4Hr1G073840 | 3-ketoacyl-CoA synthase 11 | |
| HORVU1Hr1G092240 | Glucan endo-1,3-beta-glucosidase 13 | |
